# A Review of Its Medical Aspects.—III

**Published:** 1909-03-13

**Authors:** 


### THE POOR-LAW COMMISSION'S REPORT.

### A REVIEW OF ITS MEDICAL ASPECTS.—III.

(*Continued from page* 597.)

### THE MINORITY REPORT.

The general recommendation of the Minority is that the present Boards of Guardians be abolished, and that the services now administered by the Destitution Authorities (other than those connected with vagrants or the able-bodied) be transferred to County and County Borough Councils, strengthened in numbers as may be deemed necessary for administration through their appropriate committees. Care of the sick and permanently incapacitated, the infants under school age, and the aged needing institutional care, would be assumed by the Health Committee. Further, that a Registrar of Public Assistance be appointed by the Council (1) to keep a register of all cases in receipt of public assistance, (2) assess and recover whatever charges Parliament may decide to make, and (3) sanction the grants of home aliment proposed by the respective committees.

#### (a) *The Medical Service of the Poor Law.*

Of the 915,000 simultaneous paupers in England and Wales probably 120,000 are acutely sick, requiring not merely maintenance, but also appropriate medical treatment. During the past 70 years the influence of the Central Authority on the Boards of Guardians has, in this branch of the work, been of a two-fold character; it has attempted to restrict the area of outdoor medical relief, while striving to improve the quality of indoor medical relief. This is due to the assumption that outdoor medical relief, whilst not in itself deterrent, is an open door to other forms of pauperism, whilst indoor relief, however good in quality, will always be deterrent owing to the necessary loss of liberty. We have to record that, in the great majority of Unions, especially those of rural or semi-rural character, the Boards of Guardians have shown themselves equally impervious to both sides of the advice and injunctions of the Local Government Board.

#### (b) *Domiciliary Medical Treatment Under the Poor Law.*

There are in England and Wales 3,713 district medical officers, averaging five or six for each Union, appointed for life or during good behaviour. Subject to his being qualified and residing within the district assigned to him and to his receiving a permanent and personal appointment, the selection, remuneration, and conditions of appointment are left to the Guardians, who have usually no expert advice at their command. So long as these appointments are thrown open to private practitioners they will be taken for other reasons than the salaries offered. Everywhere, with but two or three exceptions, the whole duty is entrusted at a scanty and inclusive stipend to one of the local practitioners. The conditions under which the district medical officers carry out their duties are as unsatisfactory as their remuneration is demoralising. Wool, lint, and bandages are hardly ever supplied to them; over the great part of the country there is no district nurse, and it is rare for the Guardians to make any sort of provision for nursing the outdoor sick poor. From 1834 to the present this Poor-law medical service, which costs in salaries, dispensaries, and drugs alone nearly £500,000 annually, is left without official recognition and supervision, the 3,713 district medical officers being thus deprived of such stimulus and encouragement.

There is a certain dissatisfaction with the Poor-law outdoor medical service; but the complaints do less than justice to the district medical officers themselves. We are impressed with the zeal and devotion displayed and the large amount of unpaid, unrecognised, and unrecorded work performed by them as a class. But it is not regarded as any part of the duty of the officer to insist on, or even inculcate, the personal habits necessary for recovery. It is not regarded as any part of his duty to take any steps to prevent the disease recurring or spreading. From first to last the outdoor medical service of the poor has no conception of the public health point of view.

#### (c) *The Restriction of Domiciliary Medical Treatment.*

The lavish and indiscriminate grant of medical orders has a disastrous effect both on the quality of the service rendered and on the spirit in which it is received; it tends to make the district medical officer reluctant and suspicious, and encourages fraud and malingering.

The fact that public medical aid is associated with the Poor Law is itself a deterrent, and in some Unions other deterrents are added by the way in which medical orders have to be obtained. Testimony given by a special medical investigator, and those officially responsible for the public health, makes it clear that a "deterrent" administration of Poor-law medical relief has a gravely adverse effect on the public health. Cases of infectious disease may go unrecognised, and thus restriction of medical relief does not make things easier for those responsible for the health of the district. Nor is the danger confined to the notifiable zymotic diseases. Phthisis is a disease to which one-seventh of the total cost of the Poor Law is said to be directly or indirectly due. In the early stages, when it is often curable, sufferers are not encouraged to present themselves for treatment, and because they are still capable of going to work are not technically eligible for a medical order.

#### (d) *The Hospital Branch of the Poor Law.*

Two-thirds of the sick now receiving institutional treatment at the hands of the Destitution Authority are still in the general mixed workhouses. We regret to have to report, after considering all the evidence, that the continued retention in the general mixed workhouses—never even contemplated by the Report of 1834—of a large number of sick persons requiring curative treatment, amounts at the present time to a grave public scandal.

The small rural workhouses, of which there are about 300, with fewer than 50 sick beds in each, are, over nearly half the superficial area of England and Wales, the only institutions in the nature of a hospital available. Most of these are old and ill-adapted to meet modern requirements, but the worst defects centre round the medical attendance and nursing.

With regard to the former we desire to bring no accusation against a hardworked and ill-remunerated class of doctors. Their stipends are, in the majority of cases, miserably inadequate, and with the meagre remuneration goes the system—in the rural workhouses, still, we are informed, almost universal—of requiring him to provide all the medicines he prescribes.

In spite of all the efforts of the Local Government Board there are still many rural workhouses without even a trained nurse, and no nurse, trained or untrained, for night-duty. Our Medical Investigator reported that in only two or three rural workhouses was the staff sufficient.

The general mixed workhouse is, in fact, shunned as long as possible, to the grave detriment of public health. Any suggestion for entrusting to the Destitution Authority powers of compulsory removal to such places as we have described appears to us to be out of the question.

We do not wish to suggest that what we have said applies to the 300 general mixed workhouses of the urban or more populous Unions. Among these can be found every possible variety in the character and efficiency of their provision for the sick. There has been, under steady pressure from the Central Authority, a steady process of improvement going on for the past 40 years; but all seem to us, from the standpoint of curative treatment of the sick, to suffer from the blight of being, and being felt to be, pauper establishments.

Poor-law Infirmaries form the most extreme development of the hospital branch of the Poor Law, but so far as the acute and surgical cases are concerned there is still inadequacy of medical attendance and nursing. We are inclined to attribute this not only to inadequate salaries, but even more to the lack of such *stimuli* as the supervision and inspection of the medical profession and their divorce from the work of the Public Health Authorities. They afford a striking contrast to a good London hospital in their lack of specialism, which is, if anything, officially discouraged, in case it would be a temptation to a man to come to the Poor Law. They are growing rapidly in popularity and are fast becoming rate-aided hospitals.

(e) *The Defects of the Poor-law Medical Service Traced to Their Root.*

While there is a remarkable lack of uniformity between district and district in the treatment accorded to the sick poor, the worst feature is the deterrent aspect which the medical branch of the Poor Law acquires through its association with the Destitution Authority—a feature demonstrated to us beyond all dispute. The "medical relief" given is too unconditional in character. The Poor Law spends £4,000,000 annually in relieving the present troubles of the sick, but from one end to the other of its medical service there is a complete ignoring of the preventive aspect of State medicine.

We cannot therefore recommend any extension of public medical service by the Destitution Authority, for the improvements which are needed would encourage persons to qualify for its receipt by becoming paupers, while the much-needed power of compulsory removal to institutions could scarcely be entrusted to an authority dominated by the idea of "relieving destitution."

(f) *The Treatment of the Sick by Voluntary Agencies.*

The treatment afforded by the out-patient departments of the great voluntary hospitals is, from the standpoint of preventive, or really curative treatment, wholly unsatisfactory. However genuinely useful such a department may be for a preliminary diagnosis, which promptly sifts out and admits the cases requiring institutional treatment, it still remains "to a great extent a shop for giving people large quantities of medicine." Free dispensaries and "medical missions" have all the drawbacks and none of the advantages of an out-patient department. All these centres, where the gathering of sick persons in halls or passages is involved, should be inspected to see that they are not spreading more disease than they are curing.

Speaking broadly, the friendly societies do not provide medical assistance for any women or for children; nor do they, if they can help it, admit "bad lives," nor provide it for members suffering from venereal disease or the results of alcoholic excess. Private medical clubs do provide for children and wives, though midwifery is not included; otherwise much the same objections apply. In both cases the doctor is overworked and poorly remunerated, and the overworked club doctor has little time for domiciliary work. To quote the words used by a medical witness, himself a Poor-law Guardian, "the clubs are a failure, both for the patients and for the medical men."

Provident medical associations differ only in the fact that all the medical men of the locality who are willing to take part share in the practice and in the contributions in exact proportion to the number of members who select them for their doctor. But there are many objections to the proposal to reorganise medical assistance on a provident basis. The so-called "self-supporting" agencies are partly supported by the rates and partly by private charity, so far as institutional treatment is concerned. If persons who have not paid fees are to have the fees paid for them and still have the free choice of doctors, then the strongest inducement to provide for one's own doctoring is thereby removed. These agencies take no supervision of the general hygiene, and do not provide food and nourishment. These last are frequently necessary additions to medical attendance, and the doctor most addicted to ordinary extras will secure the most patients and the largest remuneration. Such a system would end in medical *relief*, in the literal sense of the words, and that wholly without conditions.

Voluntary hospitals like to deal with the acute and interesting case; the chronic or incurable one means a transfer to the workhouse or Poor-law infirmary. They are not distributed geographically, and the selection of diseases admitted for treatment is, alike from the standpoint of preventive medicine and from that of the needs of the poor, equally arbitrary.

(g) *The Treatment of the Sick by the Public Health Authorities.*

We have in every part of the United Kingdom two public medical authorities legally responsible for, and in many cases simultaneously treating, the same class of poor persons, sometimes even for the same diseases. There have been various developments of the Public Health Service. (1) The Public Health Authorities now maintain over 700 permanent municipal hospitals, having altogether nearly 25,000 beds. The Public Health Acts do not prescribe the kind of disease to be treated in the hospital which they authorise. That their power to provide hospitals is limited to contagious or infectious disease is a mistaken assumption. In 1900 Barry started a free municipal hospital exclusively for non-infectious cases, and principally for accidents, and Widnes also started an accident hospital. But the greatest recent development has been in the treatment of tuberculosis. In Scotland the Local Government Board have laid it down that it is for the Local Health and not the Poor-law Authority to treat all cases of phthisis. (2) The Local Government Board has now ordered the Poor-law Authorities everywhere to notify to the Local Health Authorities every case of phthisis observed in the pauper population. Notification, inspection, treatment of "contacts," and disinfection have been extended from disease to disease. (3) Specific remedies in certain diseases are supplied gratuitously to ensure prompt administration. Anti-toxin serum for diphtheritic cases is freely distributed by many Health Authorities. (4) Willesden has established an out-patients' department for ringworm, impetigo, scabies, and ophthalmia. Newcastle-on-Tyne Town Council subscribes 100 guineas to the Dispensary and so secures about 800 "letters." (5) Free provision for pediculosis and scabies is expressly authorised by Parliament. For pediculosis alone, the Marylebone Health Authority successfully treated 32,500 patients in seven years. (6) The system of health visiting has now been adopted in some scores of towns, and with this new departure of carrying sanitation into the home will come an appreciation by the people themselves of the value of good health. (7) Some

Public Health Authorities have begun domiciliary treatment of the adult sick by municipal home nurses. (8) The services of the medical officer of health as diagnostician are rapidly extending. (9) In cases of suspected plague or cholera, and frequently for smallpox, free lodging and an alimentary allowance is granted to "contacts." With regard to other infectious diseases the state of things is chaotic, and great hardship arises. The far more serious problem is that presented by the necessity of granting aliment to the dependents of patients admitted to the municipal hospitals or sanatoria. There does not, however, appear to be any legal limitation to the power of the Health Authorities to grant such aliment, at any rate as regards all notifiable diseases.

*The Characteristics of the Public Health Authority's Treatment of Disease.*—All treatment of the individual patient by the Local Health Authority has for its object, not the relief of immediate suffering, but the prevention of disease; and it is preventable disease which is so great a cause of unnecessary pauperism. Zymotic diseases account, probably, for only a twentieth or thirtieth of the persons ill at any one time. For purposes of prevention early diagnosis is of supreme importance, and this is the characteristic mark of the machinery of the Public Health Service, which recognises that preventable diseases are brought about by environment, by neglect of acute cases, and by bad hygienic habits of the individual himself.

(*To be continued.*)

